# Incremental predictive value of platelet parameters for preeclampsia: results from a large prospective cohort study

**DOI:** 10.1186/s12884-023-05661-y

**Published:** 2023-05-26

**Authors:** Shan-Shan Lin, Cheng-Rui Wang, Dong-Mei Wei, Jin-Hua Lu, Xiao-Juan Chen, Qiao-Zhu Chen, Xiao-Yan Xia, Jian-Rong He, Xiu Qiu

**Affiliations:** 1 Division of Birth Cohort Study, Guangzhou Women and Children’s Medical Center, Guangzhou Medical University, Guangzhou, Guangdong China; 2 Department of Women and Child Health Care, Provincial Key Clinical Specialty of Woman and Child Health, Guangzhou Women and Children’s Medical Center, Guangzhou Medical University, Guangzhou, Guangdong China; 3 Department of Clinical Laboratory, Guangzhou Women and Children’s Medical Center, Guangzhou Medical University, Guangzhou, Guangdong China; 4 Department of Obstetrics and Gynecology, Guangzhou Women and Children’s Medical Center, Guangzhou Medical University, Guangzhou, Guangdong China; 5 Department of Women’s Health, Guangdong Provincial Key Clinical Specialty of Woman and Child Health, Guangzhou Women and Children’s Medical Center, Guangzhou Medical University, Guangzhou, 510623, China; 6 Guangdong Provincial Clinical Research Center for Child Health, Guangzhou Women and Children’s Medical Center, Guangzhou Medical University, Guangzhou, 510623, China; 7 Provincial Key Laboratory of Research in Structure Birth Defect Disease and Department of Pediatric Surgery, Guangzhou Women and Children’s Medical Center, Guangzhou Medical University, Guangzhou, 510623, China

**Keywords:** Preeclampsia, Predictive value, Platelet parameter, Pregnancy, Risk factor

## Abstract

**Background:**

Platelet parameters during pregnancy were associated with the risk of preeclampsia (PE), but the predictive value of these parameters for PE remained unclear. Our aim was to clarify the individual and incremental predictive value of platelet parameters, including platelet count (PC), mean platelet volume (MPV), plateletcrit (PCT), and platelet distribution width (PDW) for PE.

**Methods:**

This study was based on the Born in Guangzhou Cohort Study in China. Data on platelet parameters were extracted from medical records of routine prenatal examinations. Receiver operating characteristic (ROC) curve was performed to analyze the predictive ability of platelet parameters for PE. Maternal characteristic factors proposed by NICE and ACOG were used to develop the base model. Detection rate (DR), integrated discrimination improvement (IDI) and continuous net reclassification improvement (NRI) were calculated compared with the base model to assess the incremental predictive value of platelet parameters.

**Results:**

A total of 30,401 pregnancies were included in this study, of which 376 (1.24%) were diagnosed with PE. Higher levels of PC and PCT were observed at 12–19 gestational weeks in women who developed PE later. However, no platelet parameters before 20 weeks of gestation reliably distinguished between PE complicated pregnancy and non-PE complicated pregnancy, with all values of the areas under the ROC curves (AUC) below 0.70. The addition of platelet parameters at 16–19 gestational weeks to the base model increased the DR for preterm PE from 22.9 to 31.4% at a fixed false positive rate of 5%, improved the AUC from 0.775 to 0.849 (*p* = 0.015), and yielded a NRI of 0.793 (*p* < 0.001), and an IDI of 0.0069 (*p* = 0.035). Less but significant improvement in prediction performance was also observed for term PE and total PE when all the four platelet parameters were added to the base model.

**Conclusions:**

Although no single platelet parameter at the early stage of pregnancy identified PE with high accuracy, the addition of platelet parameters to known independent risk factors could improve the prediction of PE.

**Supplementary Information:**

The online version contains supplementary material available at 10.1186/s12884-023-05661-y.

## Background

Preeclampsia (PE) is a pregnancy multisystemic disease, characterized by new onset hypertension accompanied by proteinuria after 20 weeks of pregnancy [1]. It is one of the leading causes of maternal and fetal morbidity and mortality [2, 3], affecting approximately 2–8% of all pregnancies [4]. The negative effects of PE are not only confined to the immediate risk to mother and child, but also the long-term health problems including cognitive impairment, atherosclerosis, cardiovascular disease and stroke later in life [5–8]. Therefore, early prediction of pregnancy complicated with PE is of great clinical significance for disease management and improvement of pregnancy outcome.

The UK National Institute for Health and Clinical Excellence (NICE) [9] and the American College of Obstetricians and Gynecologists (ACOG) [10] have proposed traditional approach to identify women with high risk of developing PE based on their medical histories and demographic features. Recently, a more effective algorithm has been developed by the Fetal Medicine Foundation (FMF) for screening high-risk PE pregnancies with the addition of mean arterial pressure (MAP), uterine artery pulsatility index (UtA-PI), serum placental growth factor (PlGF) and pregnancy-associated plasma protein-A (PAPP-A) [11, 12]. This algorithm could predicate approximately 89, 75, and 47% of pregnancies with PE that delivery < 32, < 37 and ≥ 37 weeks, respectively, at the false-positive rate (FPR) of 10% [12], which demonstrated the superiority of combining biomarkers in PE screening. Soluble fms-like tyrosine kinase 1 (sFlt-1) is another widely proposed biomarker for the prediction of PE [13]. However, the additional cost and stringent quality requirement of UtA-PI, PlGF, PAPP-A and sFlt-1 make them unfit for many developing countries with limited resources [14, 15].

Although the etiology of PE is unknown, uncontrolled platelet activation and aggregation are observed in PE [16, 17]. Independent associations between levels of platelet parameters during pregnancy and risk for PE development have been reported by previous studies [18–20]. Considering the low cost and great accessibility of routine blood test, platelet indices are proposed as new potential biomarkers for PE. However, the predictive value of platelet parameters remains uncertain [18, 21, 22]. Few studies are available on PE prediction based on platelet parameters measured before 20 weeks of gestation [23–25], and evidence from large prospective cohort studies is limited [22, 25]. In addition, whether platelet parameters have incremental value to known predictive models has not been well studied [25].

Accordingly, we performed the present study to fully evaluate the predictive value of platelet parameters during pregnancy for the risk of PE based on data from a large cohort study. Furthermore, we aimed to identify the incremental value of these indices in identifying PE individuals.

## Methods

### Study design and participants

This study was based on the Born in Guangzhou Cohort Study (BIGCS). The BIGCS recruited women attending their first routine antenatal examinations at two campuses of the Guangzhou Women and Children’s Medical Centre (GWCMC) in Guangzhou, China from February 2012. The protocol of BICGS has been previously published [26]. Follow-ups are conducted by questionnaires at regular intervals and clinical information is obtained through medical records. The protocols of the BIGCS were reviewed and approved by the Institutional Ethics Committee of the GWCMC. Written informed consents were obtained from all participants.

**Fig. 1:**
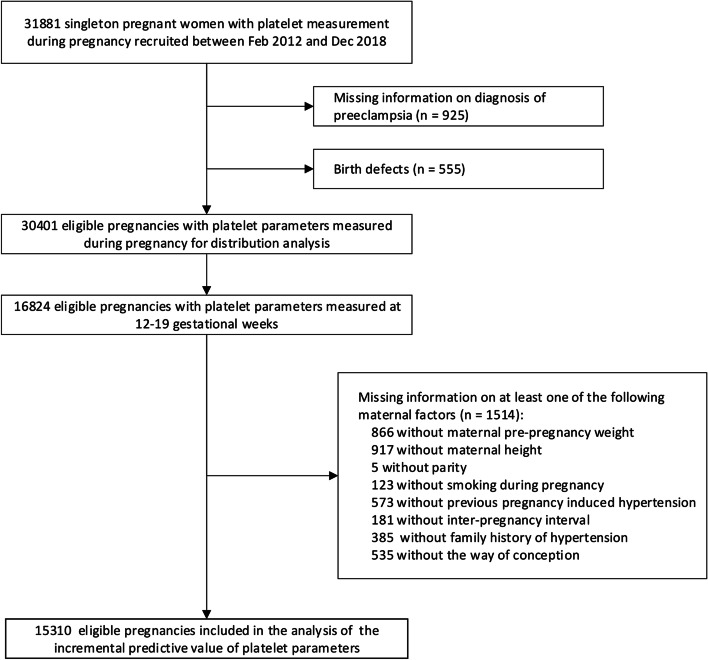
Participant selection flowchart

From Feb 2012 to Dec 2018, 31,881 singleton pregnant women with at least one platelet parameters measured during pregnancy were enrolled. Among these women, 1480 (4.6%) were excluded for missing information on PE diagnosis (*n* = 925) or with fetus diagnosed with birth defects (*n* = 555). 30,401 were included in the analysis of distributions of platelet parameters among women with and without PE at different periods of pregnancy (< 8, 8–11, 12–15, 16–19, 20–23, 24–27, 28–31, 32–35, and ≥ 36 weeks of gestation). As PE is defined as a condition that develops for the first time after 20 weeks of gestation, a subpopulation of 15,310 women with platelet parameters measured at 12–19 gestational weeks and with complete information on maternal characteristics were further included for the evaluation of incremental value of platelet parameters in predicting PE (Fig. 1).

### Maternal platelet parameters

Data on platelet parameters were obtained from laboratory information system. Routine blood tests were taken during prenatal examinations, with 2 mL of venous blood obtained by antecubital venipuncture with collection tubes containing EDTA. Platelet parameters including platelet count (PC), mean platelet volume (MPV), plateletcrit (PCT), and platelet distribution width (PDW) were measured with the automated blood cell counter (Sysmex XE-5000, Sysmex, Kobe, Japan) within 2 h of sampling. All blood samples were collected, handled and processed in the same way by trained medical personnel. The first test record of platelet parameters was used in analysis when there was more than one test available in a certain pregnancy period.

### Definition of PE

The presence of doctor-diagnosed PE was retrieved from medical records. Diagnosis of PE was according to the International Society for the Study of Hypertension in Pregnancy [27], considered: systolic blood pressure > 140 mmHg, and/or the diastolic blood pressure > 90 mmHg on at least two occasions, 4 h apart, developing after 20 weeks of gestation in previously normotensive women and proteinuria > 300 mg in 24 h or two readings of at least + + on dipstick analysis of midstream or catheter urine specimens, whenever no 24-h collection is available. Women with chronic hypertension that developed significant (as defined above) proteinuria after 20 weeks of gestation, were classified as having superimposed PE. Preterm PE and term PE were defined as PE with delivery at < 37 and ≥ 37 weeks of gestation, respectively.

### Maternal risk factors

According to NICE and ACOG [9, 10], the available maternal predictors in the present study to develop the base models for PE predication including maternal age (continuous), pre-pregnancy weight (continuous), height (continuous), cigarette smoking during pregnancy (yes or no), parity (multiparous or nulliparous), inter-pregnancy interval (continuous), way of conception (spontaneous or in-vitro fertilization), history of chronic hypertension (yes or no), history of pre-existing diabetes mellitus (yes or no), family history of chronic hypertension (yes or no). We did not collect information on history of previous PE, but history of previous pregnancy induced hypertension (PIH) was used instead in this study. Risk factors including history of chronic kidney diseases and systemic lupus erythematosus or anti-phospholipid syndrome were not included as few women with these diseases in this population (0.02% and 0.04%, respectively). The gestational age used in this study was confirmed by ultrasound measurement at first or second trimester. Data on maternal characteristics and medical history was obtained by self-administered questionnaires at recruitment and from the medical records.

### Statistical analysis

Demographic and clinical data of the participants were described as mean ± standard deviation (SD) for continuous variables and as number and percentage for categorical variables. The distributions of platelet parameters between women with and without PE at different periods of pregnancy were compared with Kruskal–Wallis test. The association between platelet parameters with the risk of PE was modeled with a restricted cubic spline to explore the potential linear or non-linear relationships. Platelet parameters were fitted in the regression models as continuous variables. The test for overall association was checked by testing that both the coefficients associated with the linear and the non-linear components are equal to zero (the null hypothesis being no association between platelet parameters with the risk of PE). If we fail to reject the hypothesis, the non-linear association was tested. The numbers and placements of knots for restricted cubic spline analysis were chosen based on the Akaike’s information criterion.

We constructed receiver operating characteristic (ROC) curves for each platelet parameter to evaluate the prediction performance. Areas under the ROC curves (AUC), sensitivity and specificity for each platelet parameter were calculated for PE prediction. The optimal cut-off value was defined as the point with the highest sum of sensitivity and specificity. Detection rate (DR) was evaluated at a FPR of 5% and 10%. And integrated discrimination improvement (IDI) and category-free net reclassification improvement indices (NRI) were calculated with logistic regressions to quantify the added value of platelet parameters to that of the base models. The numerical platelet parameters were categorized as binary variables based on the best cut-off value points defined by ROC curve analysis when fitted in the logistic models. IDI and NRI offer additional information regarding the incremental yield of a new biomarker over the area under the ROC curve [28]. The continuous NRI is a measure of improvement in reclassification. Defining PE as the event, it was described as the sum of NRI_(events)_ and NRI_(non-events)_ and was interpreted as the proportion of women reclassified to a more appropriate risk on addition of the platelet parameters to the logistic regression [29]. In women who developed PE, if the addition of the platelet parameters resulted in more individuals being reclassified to a higher risk, then the NRI_(events)_ was positive. For women who did not develop PE, if more women were assigned as lower risk, then the NRI_(non-events)_ was positive. IDI was defined as the average increase in predicted risk of PE in women with PE added to the average decrease in predicted risk in women without PE [29].

The statistical software package SAS, version 9.4 and R, version 4.2.3 was used for data analyses. All statistical tests were two-sided and used an α of 0.05 as the threshold of statistical significance.

## Results

A summary of maternal characteristics (demographic, anthropometric, and medical history) are presented in Table 1. Of the 30,401 pregnancies, 376 (1.24%) women were diagnosed with PE. Compared to women in the non-PE group, women who developed PE were older, with higher pre-pregnancy BMI, longer inter-pregnancy interval, more likely to have previous PIH, chronic hypertension and family history of chronic hypertension, and more likely to be conceived by in-vitro fertilization (*p* for all < 0.01).

**Table 1:** Characteristics of pregnant women with and without preeclampsia

Characteristics	Non-preeclampsia pregnancy (*n* = 30025)	Preeclampsia pregnancy (*n* = 376)	*p-*value
**Maternal age at conception, years, mean ± SD**	30.1 ± 3.86	31.2 ± 4.14	< 0.0001
**Maternal Pre-pregnancy weight, kg, mean ± SD**	52.7 ± 7.63	57.1 ± 10.12	< 0.0001
**Maternal height, cm, mean ± SD**	159.9 ± 5.01	159.5 ± 5.27	0.237
**Pre-pregnancy BMI, kg/m**^**2**^**, mean ± SD**	20.6 ± 2.73	22.4 ± 3.77	< 0.0001
**Smoke during pregnancy, n (%)**	175 (0.6)	4 (1.1)	0.221
**Parity**			
Nulliparous, n (%)	20477 (68.2)	269 (71.5)	0.170
Parous with previous PIH, n (%)	93 (0.3)	8 (2.2)	< 0.0001
Pregnancy interval in years, mean ± SD	5.7 ± 3.27	6.9 ± 3.75	0.001
**Medical history**			
Chronic hypertension, n (%)	119 (0.4)	7 (1.9)	< 0.0001
Diabetes mellitus, n (%)	111 (0.4)	2 (0.5)	0.608
**Family history of chronic hypertension, n (%)**	7986 (27.2)	154 (41.7)	< 0.0001
**Conception**			0.003
Spontaneous, n (%)	28228 (97.0)	344 (94.3)	
In-vitro fertilization, n (%)	880 (3.0)	21 (5.8)	

*Abbreviations*: *n* number, *SD* standard deviation, *PIH* pregnancy induced hypertension

**Fig. 2:**
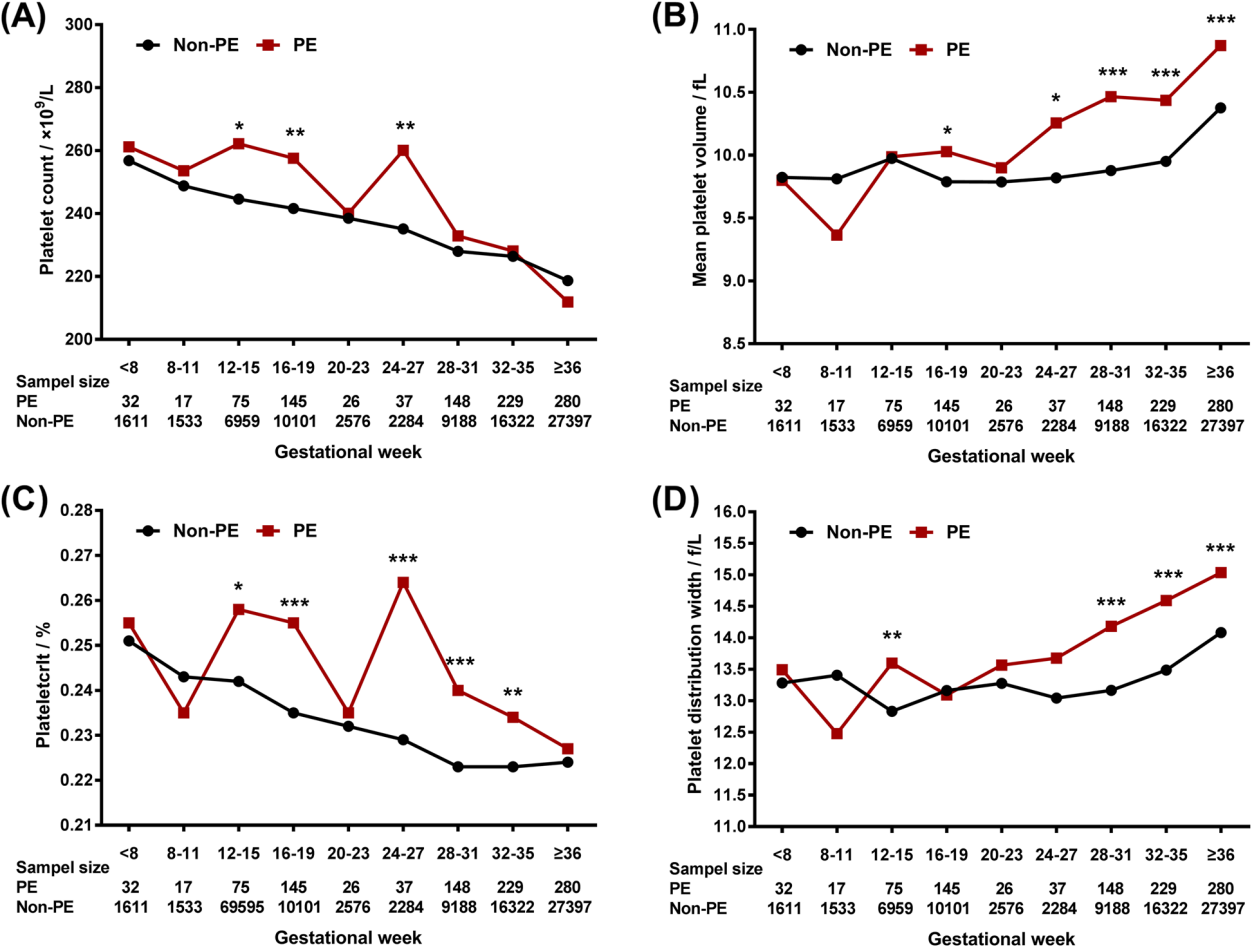
Distributions of platelet parameters during pregnancy among pregnant women with and without preeclampsia. The distributions of platelet parameters between women with and without PE were compared with Kruskal–Wallis test for each period separately. ^*^ *p* < 0.05, ^**^ *p* < 0.01, ^***^ *p* < 0.001, compared to women without preeclampsia. PE, preeclampsia

The distributions of platelet parameters throughout pregnancy among women with and without PE are shown in Fig. 2 and Table S1. No significant differences between the two groups were observed in the first trimester. Women with PE had higher means of PC at 12–15, 16–19 and 24–27 gestational weeks (*p* for all < 0.05). While for MPV, before 20 weeks of gestation, the difference between PE and non-PE group was not significant except for 16–19 weeks of gestation (*p* < 0.05), after which the differences between groups began more obvious. The levels of PCT were significantly higher among pregnancies complicated with PE at 12–15, 16–19 and 24–36 gestational weeks than non-PE group (*p* for all < 0.05). Significantly higher levels of PDW were observed in PE group at 12–15 and > 28 weeks of gestation (*p* for all < 0.05).

As PE was firstly diagnosed after 20 weeks of gestation, and based on the distributions of platelet parameters among women with and without PE, the predictive values of each platelet parameter at 12–15 and 16–19 gestational weeks were further analyzed. A linear association with PE was found for PC (*p* for linearity = 0.0044) and PDW (*p* for linearity = 0.0391) at 12–15 gestational weeks, and for PC (*p* for linearity = 0.0031) and PCT (*p* for linearity = 0.0003) at 16–19 gestational weeks (Fig. S1 and S2). As shown in Table 2, there was not a threshold for these platelet parameters at which PE could be reliably predicted (AUC < 0.70 for all platelet parameters). The predictive ability was similar when PE was further classified as preterm PE and term PE. PCT measured at 16–19 gestational week had a highest sensitivity of 0.892 for preterm PE, while the specificity was 0.449. The ROC analysis for platelet parameters on PE prediction during other periods of pregnancy are presented in Table S2.

**Table 2:** Diagnostic performance of platelet parameters for preeclampsia based on receiver-operating characteristic analysis

	**12–15 weeks of gestation (*n* = 7034)**				**16–19 weeks of gestation (*n* = 10,246)**			
	**PC**	**MPV**	**PCT**	**PDW**	**PC**	**MPV**	**PCT**	**PDW**
**Total PE**								
AUC	0.580	0.515	0.590	0.580	0.580	0.559	0.608	0.499
Sensitivity	44.0	48.0	77.3	60.5	65.5	54.5	71.7	75.2
Specificity	72.8	60.8	38.8	54.4	47.7	57.6	45.0	31.0
**Preterm PE**								
AUC	0.543	0.596	0.516	0.597	0.616	0.612	0.688	0.552
Sensitivity	55.0	44.0	35.0	45.0	37.8	45.9	89.2	83.8
Specificity	67.8	83.8	76.0	83.0	82.9	74.3	44.9	28.1
**Term PE**								
AUC	0.593	0.555	0.629	0.573	0.566	0.540	0.580	0.520
Sensitivity	43.6	30.9	76.4	56.4	49.1	65.7	59.3	95.4
Specificity	72.7	83.1	46.4	57.8	63.4	44.2	52.9	13.2

*Abbreviations*: *PE* preeclampsia, *PC* platelet count, *MPV* mean platelet volume, *PCT* plateletcrit, *PDW* platelet distribution width, *AUC* areas under the ROC

The incremental prediction values of platelet parameters at 16–19 gestational weeks to the base model are summarized in Tables 3 and 4. The DRs for preterm PE, term PE and total PE were 22.9%, 17.9% and 19.2% at a 5% FPR, respectively based on maternal characteristics. The addition of the four platelet parameters improved the DRs to 31.4%, 23.2% and 22.3% for preterm PE, term PE and total PE respectively, and improved the AUC from 0.775 to 0.849 (preterm PE, *p* = 0.015), 0.674 to 0.701 (term PE, *p* = 0.021), and 0.694 to 0.717 (total PE, *p* = 0.015), respectively. In addition, the NRIs for preterm PE, term PE and total PE were 0.793 (*p* < 0.001), 0.254 (*p* = 0.014) and 0.178 (*p* = 0.043), the IDIs were 0.0069 (*p* = 0.035), 0.0022 (*p* = 0.001) and 0.0020 (*p* = 0.007) with the addition of the four platelet parameters, respectively. The corresponding analyses for platelet parameters measured at 12–15 gestational weeks are shown in Table S3-4. For platelet parameters measured at 12–15 weeks of gestation, although improvements in DRs were observed, AUCs were not substantially increased by adding individual or combined platelet parameters.

**Table 3:** Detection rates for preeclampsia < 37, ≥ 37 weeks of gestation, and for all preeclampsia for 5% and 10% false-positive rates based on maternal characteristics and platelet parameters at 16–19 gestational weeks (*n* = 9346)

	**Preterm PE**				**Term PE**				**Total PE**			
	**AUC**	***p*-value**	**Detection rate (95%)**		**AUC**	***p*-value**	**Detection rate (95%)**		**AUC**	***p*-value**	**Detection rate (95%)**	
			**FPR 5%**	**FPR 10%**			**FPR 5%**	**FPR 10%**			**FPR 5%**	**FPR 10%**
Base model	0.775		22.9 (8.6–37.1)	45.7 (28.6–62.9)	0.674		17.9 (10.5–27.4)	34.7 (25.3–44.2)	0.694		19.2 (12.3–27.7)	37.7 (29.2–46.2)
Plus												
PC	0.786	0.332	20.0 (8.6–34.3)	40.0 (22.9–57.1)	0.683	0.184	17.9 (10.5–26.3)	36.8 (27.4–46.3)	0.706	0.018	21.5 (13.9–29.2)	35.4 (26.9–43.9)
MPV	0.807	0.158	34.3 (17.1–51.4)	48.6 (31.4–65.7)	0.677	0.639	21.1 (12.6–31.6)	34.7 (25.3–45.3)	0.700	0.445	21.5 (14.6–29.2)	38.5 (30.6–47.7)
PCT	0.821	0.066	25.7 (11.4–40.0)	42.9 (25.7–60.0)	0.679	0.366	19.0 (11.6–27.4)	34.7 (26.3–45.3)	0.711	0.041	21.4 (13.9–29.2)	33.9 (26.2–43.1)
PDW	0.772	0.704	22.9 (11.4–37.1)	42.9 (28.6–60.0)	0.687	0.168	22.1 (12.6–31.6)	34.7 (25.3–44.2)	0.696	0.539	20.8 (13.9–27.7)	36.2 (27.7–43.9)
PC + MPV + PCT + PDW	0.849	0.015	31.4 (17.1–48.6)	48.6 (31.4–65.7)	0.701	0.021	23.2 (14.7–32.6)	34.7 (25.3–45.3)	0.717	0.015	22.3 (14.6–30.0)	38.5 (29.2–46.9)

Multivariate logistic regression models were used to develop the base models with the recognized historic risk factors recommended by NICE and ACOG, including maternal age (continuous), pre-pregnancy weight (continuous), height (continuous), cigarette smoking during pregnancy (yes or no), parity (multiparous or nulliparous), inter-pregnancy interval (continuous), way of conception (spontaneous or in-vitro fertilization), history of chronic hypertension (yes or no), history of pre-existing diabetes mellitus (yes or no), family history of chronic hypertension (yes or no)

*PE* preeclampsia, *PC* platelet count, *MPV* mean platelet volume, *PCT* plateletcrit, *PDW* platelet distribution width, *AUC* areas under the ROC

**Table 4:** The incremental value of platelet parameters at 16–19 gestational weeks for preeclampsia prediction (*n* = 9346)

	**NRI**	***p-* value**	**Event NRI**	***p-* value**	**Non-Event NRI**	***p-* value**	**IDI**	***p-* value**	**Relative IDI (%)**
Total PE (*n* = 130)									
Base model	Ref		Ref		Ref		Ref		Ref
Base model + PC	0.240	0.007	0.28	0.002	-0.04	< 0.001	0.0001	0.859	0.51
Base model + MPV	0.216	0.014	0.06	0.483	0.15	< 0.001	0.0016	0.016	11.72
Base model + PCT	0.321	< 0.001	0.42	< 0.001	-0.09	< 0.001	0.0012	0.018	8.56
Base model + PDW	0.106	0.231	0.46	< 0.001	-0.36	< 0.001	0.0004	0.107	2.77
Base model + PC + MPV + PCT + PDW	0.178	0.043	0.08	0.381	0.10	< 0.001	0.0020	0.007	14.77
Preterm PE (*n* = 35)									
Base model	Ref		Ref		Ref		Ref		Ref
Base model + PC	0.341	0.044	-0.31	0.063	0.66	< 0.001	0.0003	0.801	2.51
Base model + MPV	0.462	0.006	-0.03	0.866	0.49	< 0.001	0.0054	0.190	42.81
Base model + PCT	0.675	< 0.001	0.77	< 0.001	-0.10	< 0.001	0.0025	< 0.001	19.44
Base model + PDW	0.173	0.306	0.60	< 0.001	-0.43	< 0.001	0.0006	0.025	4.89
Base model + PC + MPV + PCT + PDW	0.793	< 0.001	0.43	0.011	0.36	< 0.001	0.0069	0.035	54.48
Term PE (*n* = 95)									
Base model	Ref		Ref		Ref		Ref		Ref
Base model + PC	0.240	0.020	-0.03	0.758	0.27	< 0.001	0.0002	0.606	1.93
Base model + MPV	0.149	0.148	0.26	0.010	-0.11	< 0.001	0.0009	0.031	9.82
Base model + PCT	0.201	0.051	0.14	0.182	0.06	< 0.001	0.0003	0.252	2.97
Base model + PDW	0.160	0.120	0.89	< 0.001	-0.73	< 0.001	0.0015	< 0.001	16.22
Base model + PC + MPV + PCT + PDW	0.254	0.014	0.16	0.124	0.10	< 0.001	0.0022	0.001	23.16

Multivariate logistic regression models were used to develop the base models with the recognized historic risk factors recommended by NICE and ACOG, including maternal age (continuous), pre-pregnancy weight (continuous), height (continuous), cigarette smoking during pregnancy (yes or no), parity (multiparous or nulliparous), inter-pregnancy interval (continuous), way of conception (spontaneous or in-vitro fertilization), history of chronic hypertension (yes or no), history of pre-existing diabetes mellitus (yes or no), family history of chronic hypertension (yes or no)

*PE* preeclampsia, *PC* platelet count, *MPV* mean platelet volume, *PCT* plateletcrit, *PDW* platelet distribution width, *NRI* net reclassification improvement, *IDI* integrated discrimination improvement

## Discussion

This study based on a large, prospectively enrolled cohort of pregnant women comprehensively evaluated the individual and incremental predictive values of platelet parameters in PE. The analysis of our data demonstrated that although individual platelet parameters had limited discrimination ability in identifying which pregnant women would develop PE, platelet indices at the early stage of pregnancy had incremental value in predicting PE when added to known independent risk factors using various statistical analyses.

Few studies have fully compared the levels of platelet parameters between women with and without PE throughout pregnancy [30–32]. Our findings from a very large number of populations showed comparable levels of platelet parameters at the beginning of pregnancy among women with and without PE. While at the end of the first trimester, higher platelet parameters tended to show in women who developed PE later. The pathogenesis of PE is characterized by endothelial damage in the spiral arteries during placentation in the first trimester. Our data also supported that platelet dysfunction in PE complicated pregnancy started as soon as early pregnancy. A speeder decrease rate in PC and PCT and a quicker increase in MPV occurred from the mid-later trimester among women with PE was observed in our population, which was supported by previous study [30, 32]. The differences in the trajectories of platelet parameters during pregnancy between women with and without PE highly indicated a role of platelets in PE pregnancy. However, data from the present study could not demonstrate the causal relationship between altered platelet function and the development of PE. Further studies were required to clarify the precise contribution of platelets in the pathogenesis of PE.

Strong evidence was lacking on the prediction performance of platelet parameters for PE. The results of most previous studies were based on data with platelet parameters collected from the late pregnancy and remained conflicted [18, 23, 24, 33–35]. One prospective study with 9552 participants showed a relatively good prediction of second trimester MPV for PE, with a sensitivity of 95.2% and a specificity of 66.7% [22]. However, other authors did not confirm this performance, with the reported AUCs of platelet parameters at early or mid-pregnancy below 0.70 [21, 36, 37]. A recent small nested case–control study reported that PLT/MPV was a good predictor for PE, with sensitivity of 83.7% and specificity of 86.2% [38]. However, the performance of this ratio in our population was poor (data not shown). Our study extended previous researches with the evaluation of four platelet parameters at different stages of pregnancy in PE prediction, and reinforced this evidence that the discrimination ability of individual platelet parameter during each periods of pregnancy in identifying PE was low.

For the prediction of high-risk pregnant women for PE, the FMF proposed a new algorithm using a competing risk model to estimate a priori risk of patient-specific risk of PE [11, 12]. And they showed an approximately 10% higher DR than that of the NICE guidelines [9]. In the present study, we used multivariable logistic regression models to estimate the risk of PE based on maternal characteristics and medical history, which showed a DR of 22.9, 17.9 and 19.2% for preterm PE, term PE and total PE at a 5% FPR, respectively. However, the reported DRs were ranged from 28.0–47.6% for preterm PE, 24–30% for term PE, and 26–40.3% for total PE, which were relatively higher than our results [9, 11, 39–43]. It is reasonable to hypothesize that the use of competing risk model for a priori risk might improve the DR of our study. However, it is also true that the performance of PE prediction models is partly population dependent. Most of the previous studies were conducted in Europeans and North Americans, with a higher incidence of PE than that of our population [39]. The results from two studies conducted in Asian population showed a comparable DR with our findings [44, 45], which supported the reliability of our data.

A single parameter might be limited in its ability to predict responses accurately because multivariable factors affect the response [46]. Several metrics are proposed to quantify the incremental utility of a candidate biomarker or a test, including ΔAUC, NRI, and IDI. ΔAUC has been widely used [47], and NRI and IDI are proposed to assess overall improvement of reclassifications [28]. Few studies formally quantified the incremental value of platelet indices in PE prediction. The results from a large cohort study in China showed that the combination of PC tested at 5–10 and 19–23 weeks of gestation could further improve the prediction performance of models based on demographical characteristics and mean arterial pressure for PE (AUC from 0.76 to 0.79) [25]. However, the incremental predictive values of platelet parameters measured at other stages were not evaluated, nor the predictive values of other platelet parameters. Our large analysis based on data of four platelet parameters measured during the whole pregnancy indicated that the inclusion of the four platelet parameters had the highest incremental predictive value for PE. In addition, the significant event-NRI obtained in the present study indicated that the new model overall performed best at correctly identifying individuals with PE. However, given the low number of PE cases in the present study, our results should be interpreted with caution.

The better screening efficacy for preterm PE than term PE observed in the present study has also been reported in other models with different predictors [12, 31, 40, 42]. This may indicate different etiological mechanisms between term PE and preterm PE. Recent studies on placental pathology indicated that early-onset PE was associated with abnormal placental morphology, while placentas from late-onset PE were morphologically similar to placentas from matched control [48]. A nested case–control study evaluating the role of adiponectin and insulin resistance in PE development also supported a different pathogenesis between early and late-onset PE [49]. In addition, data of uterine artery Doppler indices in the first trimester suggested that preterm PE was strongly associated with defective invasion of the spiral arteries, in contrast to the findings in term PE which may be a consequence of placental deterioration at term [50].

Although the effectiveness of models based on the combination of maternal characteristics plus biophysical parameters and biochemical biomarkers, including UtA-PI, PlGF, PAPP-A and sFlt-1, in PE prediction have been proven, many of these biomarkers are currently not readily available in economical restricted countries. And the additional cost, specific training, standardized techniques and quality control required by these biomarkers further restricted the feasibility of these biomarkers. In addition, the universal application of these biomarkers might not be appropriate in population with low incidence of PE, from economical perspectives. On the contrary, our study demonstrated that platelet parameters had incremental value in PE prediction in a low-risk population, which incurs no additional costs as part of the complete blood count with great accessibility. For resource limited areas or low-risk population, the addition of platelet parameters in the first step screening of PE might be a potential option, which would help improve risk stratification of PE at a low health economic cost.

The strengths of the present study were that, first, it was a prospective study with a complete follow-up in an unselected population. Second, the predictive abilities of platelet parameters during the whole pregnancy were comprehensively evaluated to find the optimal indices and periods for PE screening. In addition, it supplemented the data of incremental value of platelet parameters in the clinical use of PE screening. Our study had several limitations. As most of the pregnant women were recruited around 16 weeks of gestation, our analysis of platelet parameters measured in the first trimester for PE prediction was limited by a small number of populations. And we also did not validate the accuracy of the platelet parameters measured with the automated blood cell counter. However, 2.4% of pregnant women in our cohort had more than one set of platelet parameters measurements at 16–19 gestational weeks, and the data showed a considerable intra-individual stability (Table S5), which has also been reported by previously studies [46, 47]. In addition, the prevalence of PE in our population was lower than that has been reported. Thus the generalizability of our results to other population remained uncertain.

## Conclusions

Based on an unselected population, our study demonstrated an incremental but not independent value of platelet parameters at the early second trimester in identifying PE when added to other known predictors. Given the low cost and high accessibility, platelet parameters might be potential biomarkers for the clinical use for PE prediction. However, further study to verify the efficacy in other population is warranted.

## Supplementary Information

**Additional file 1:** **Table S1.** Distribution of platelet parameters throughout pregnancy among women with preeclampsia or without preeclampsia. **Table S2.** Diagnostic performance of platelet parameters during pregnancy for the detection of preeclampsia based on receiver-operating characteristic analysis. **Table S3.** Detection rates for preeclampsia＜37, ≥37 weeks of gestation, and for all preeclampsia for 5 and 10% false-positive rates based on maternal characteristics and platelet parameters 12-15 gestational weeks (*n*=6381). **Table S4.** The incremental value of platelet parameters at 12-15 gestational weeks for preeclampsia prediction (*n*=6381). **Table S5.** Platelet parameters of pregnant women with multiple measurements during 16-19 gestational weeks. **Figure S1.** Associations between platelet parameters during 12-15 weeks of gestation and preeclampsia with a restricted cubic spline function. **Figure S2.** Associations between platelet parameters during 16-19 weeks of gestation and preeclampsia with a restricted cubic spline function.

## Data Availability

The datasets used and/or analysed during the current study are available from the corresponding author on reasonable request.

## List of abbreviations

ACOG: The American Congress of Obstetricians and Gynecologists
aOR: Adjusted odds ratio
AUC: Area under the curve
BICGS: Born in Guangzhou Cohort Study
BMI: Body mass index
DR: Detection rate
FMF: The Fetal Medicine Foundation
FPR: False-positive rate
GWCMC: Guangzhou Women and Children’s Medical Centre
IDI: Integrated discrimination improvement
MAP: Mean arterial pressure
MPV: Mean platelet volume
NICE: The UK National Institute for Health and Clinical Excellence
NRI: Net reclassification improvement
OR: Odds ratio
PAPP-A: Pregnancy-associated plasma protein-A
PC: Platelet count
PCT: Plateletcrit
PDW: Platelet distribution width
PE: Preeclampsia
PIH: Pregnancy induced hypertension
PlGF: Serum placental growth factor
ROC: Receiver operating characteristic
sFlt-1: Soluble fms-like tyrosine kinase 1
SD: Standard deviation
UtA-PI: Uterine artery pulsatility index
