# Supplementary material for: Incremental predictive value of platelet parameters for preeclampsia: results from a large prospective cohort study
Source: BMC Pregnancy Childbirth. 2023 May 26;23:387. doi: 10.1186/s12884-023-05661-y (PMC10214644; doi:10.1186/s12884-023-05661-y)
Supplement: Supplementary file 1 — Additional file 1: Table S1. Distribution of platelet parameters throughout pregnancy among women with preeclampsia or without preeclampsia. Table S2. Diagnostic performance of platelet parameters during pregnancy for the detection of preeclampsia based on receiver-operating characteristic analysis. Table S3. Detection rates for preeclampsia＜37, ≥37 weeks of gestation, and for all preeclampsia for 5 and 10% false-positive rates based on maternal characteristics and platelet parameters 12-15 gestational weeks (n=6381). Table S4. The incremental value of platelet parameters at 12-15 gestational weeks for preeclampsia prediction (n=6381). Table S5. Platelet parameters of pregnant women with multiple measurements during 16-19 gestational weeks. Figure S1. Associations between platelet parameters during 12-15 weeks of gestation and preeclampsia with a restricted cubic spline function. Figure S2. Associations between platelet parameters during 16-19 weeks of gestation and preeclampsia with a restricted cubic spline function. [file 12884_2023_5661_MOESM1_ESM.docx]

**Table S1** Distribution of platelet parameters throughout pregnancy among women with preeclampsia or without preeclampsia

|  | Preeclampsia | | | |  | Non-preeclampsia | | | |
| --- | --- | --- | --- | --- | --- | --- | --- | --- | --- |
|  | PC, ×10^9^/L, Median (Quartile) | MPV, fL, Median (Quartile) | PCT, %, Median (Quartile) | PDW, fL, Median (Quartile) |  | PC, ×10^9^/L, Median (Quartile) | MPV, fL, Median (Quartile) | PCT, %, Median (Quartile) | PDW, fL, Median (Quartile) |
| <8 gw | 250.0 (82.0) | 10.25 (2.50) | 0.255 (0.070) | 12.80 (3.80) |  | 252.0 (74.0) | 9.90 (1.90) | 0.255 (0.080) | 12.80 (4.70) |
| 8-11 gw | 252.0 (80.0) | 9.70 (2.10) | 0.250 (0.090) | 11.90 (3.20) |  | 245.0 (74.0) | 9.90 (1.90) | 0.240 (0.080) | 13.00 (4.50) |
| 12-15 gw | 261.0 (91.0) | 10.20 (2.10) | 0.250 (0.060) | 13.20 (4.90) |  | 241.0 (66.0) | 10.10 (1.50) | 0.240 (0.060) | 12.30 (3.50) |
| 16-19 gw | 253.0 (80.0) | 10.20 (1.40) | 0.250 (0.070) | 12.60 (3.30) |  | 237.0 (68.0) | 9.90 (1.80) | 0.230 (0.070) | 12.60 (4.70) |
| 20-23 gw | 228.0 (56.0) | 9.90 (2.20) | 0.230 (0.050) | 13.75 (4.60) |  | 235.0 (69.0) | 9.90 (1.80) | 0.230 (0.070) | 12.70 (4.90) |
| 24-27 gw | 255.0 (72.0) | 10.40 (2.20) | 0.270 (0.080) | 13.80 (4.30) |  | 230.5 (69.0) | 10.00 (1.60) | 0.220 (0.070) | 12.35 (4.80) |
| 28-31 gw | 227.5 (84.0) | 10.55 (1.70) | 0.240 (0.080) | 13.80 (4.75) |  | 223.0 (69.0) | 10.00 (1.50) | 0.220 (0.060) | 12.50 (4.50) |
| 32-35 gw | 225.0 (84.0) | 10.40 (2.10) | 0.230 (0.080) | 14.60 (4.60) |  | 222.0 (70.0) | 10.10 (1.70) | 0.220 (0.070) | 12.90 (4.90) |
| ≥36 gw | 207.5 (81.5) | 10.90 (1.95) | 0.220 (0.080) | 14.60 (4.40) |  | 214.0 (71.0) | 10.50 (1.70) | 0.220 (0.070) | 13.60 (4.50) |

Abbreviations: preeclampsia; PC, platelet count; MPV, mean platelet volume; PCT, plateletcrit; PDW, platelet distribution width; gw, gestational weeks.

**Table S2** Diagnostic performance of platelet parameters during pregnancy for the detection of preeclampsia based on receiver-operating characteristic analysis

|  |  | Total PE | | |  | Preterm PE | | |  | Term PE | | |
| --- | --- | --- | --- | --- | --- | --- | --- | --- | --- | --- | --- | --- |
|  |  | AUC | Sensitivity | Specificity |  | AUC | Sensitivity | Specificity |  | AUC | Sensitivity | Specificity |
| <8 weeks of gestation (n=1643) | | |  |  |  |  |  |  |  |  |  |  |
|  | PC | 0.509 | 18.8 | 91.0 |  | 0.619 | 44.4 | 91.0 |  | 0.534 | 65.2 | 48.8 |
|  | MPV | 0.491 | 25.0 | 89.2 |  | 0.732 | 55.6 | 89.2 |  | 0.603 | 65.2 | 63.6 |
|  | PCT | 0.524 | 90.6 | 17.3 |  | 0.486 | 22.2 | 91.7 |  | 0.527 | 95.7 | 17.3 |
|  | PDW | 0.531 | 84.4 | 32.1 |  | 0.575 | 66.7 | 55.3 |  | 0.573 | 95.7 | 32.2 |
| 8-11 weeks of gestation (n=1550) | | |  |  |  |  |  |  |  |  |  |  |
|  | PC | 0.533 | 64.7 | 51.3 |  | 0.586 | 60.0 | 74.6 |  | 0.582 | 75.0 | 51.3 |
|  | MPV | 0.588 | 41.2 | 74.2 |  | 0.596 | 80.0 | 56.2 |  | 0.585 | 100.0 | 16.7 |
|  | PCT | 0.528 | 47.1 | 65.4 |  | 0.627 | 60.0 | 72.0 |  | 0.486 | 100.0 | 11.1 |
|  | PDW | 0.611 | 58.8 | 60.5 |  | 0.667 | 80.0 | 70.2 |  | 0.727 | 100.0 | 38.4 |
| 20-23 weeks of gestation (n=2602) | | |  |  |  |  |  |  |  |  |  |  |
|  | PC | 0.518 | 92.3 | 25.3 |  | 0.638 | 71.4 | 59.4 |  | 0.527 | 57.9 | 59.9 |
|  | MPV | 0.529 | 34.6 | 84.0 |  | 0.653 | 57.1 | 83.9 |  | 0.517 | 26.3 | 83.8 |
|  | PCT | 0.540 | 96.2 | 18.8 |  | 0.744 | 71.4 | 70.0 |  | 0.535 | 84.2 | 37.6 |
|  | PDW | 0.536 | 53.8 | 60.6 |  | 0.619 | 85.7 | 55.6 |  | 0.506 | 47.4 | 60.5 |
| 24-27 weeks of gestation (n=2321) | | |  |  |  |  |  |  |  |  |  |  |
|  | PC | 0.637 | 78.4 | 47.5 |  | 0.594 | 80.0 | 50.5 |  | 0.652 | 51.9 | 76.2 |
|  | MPV | 0.596 | 35.1 | 87.1 |  | 0.670 | 60.0 | 87.0 |  | 0.567 | 25.9 | 86.9 |
|  | PCT | 0.672 | 64.9 | 65.5 |  | 0.682 | 60.0 | 75.8 |  | 0.666 | 48.1 | 81.1 |
|  | PDW | 0.571 | 54.1 | 65.8 |  | 0.737 | 90.0 | 66.6 |  | 0.508 | 18.5 | 90.0 |
| 28-31 weeks of gestation (n=9336) | | |  |  |  |  |  |  |  |  |  |  |
|  | PC | 0.525 | 38.5 | 70.2 |  | 0.545 | 57.7 | 55.6 |  | 56.200 | 43.8 | 70.2 |
|  | MPV | 0.617 | 43.2 | 78.2 |  | 0.666 | 55.8 | 78.1 |  | 58.900 | 36.5 | 78.0 |
|  | PCT | 0.592 | 39.2 | 75.3 |  | 0.555 | 28.8 | 84.5 |  | 61.000 | 47.9 | 69.2 |
|  | PDW | 0.607 | 60.8 | 57.8 |  | 0.684 | 65.4 | 64.3 |  | 56.400 | 56.3 | 57.6 |
| 32-35 weeks of gestation (n=16551) | | |  |  |  |  |  |  |  |  |  |  |
|  | PC | 0.507 | 38.4 | 68.8 |  | 0.604 | 46.3 | 75.8 |  | 55.300 | 44.4 | 68.3 |
|  | MPV | 0.588 | 36.2 | 80.1 |  | 0.614 | 40.3 | 84.6 |  | 57.600 | 54.9 | 58.8 |
|  | PCT | 0.552 | 33.6 | 74.6 |  | 0.523 | 53.7 | 52.9 |  | 58.300 | 32.7 | 79.8 |
|  | PDW | 0.605 | 53.7 | 65.8 |  | 0.656 | 62.7 | 66.4 |  | 58.200 | 0.5 | 65.7 |
| ≥ 36 weeks of gestation (n=27677) | | |  |  |  |  |  |  |  |  |  |  |
|  | PC | 0.539 | 23.6 | 87.7 |  | 0.618 | 40.7 | 88.0 |  | 53.000 | 21.7 | 87.7 |
|  | MPV | 0.597 | 41.8 | 73.7 |  | 0.615 | 48.1 | 73.6 |  | 59.500 | 62.5 | 52.6 |
|  | PCT | 0.509 | 16.8 | 87.1 |  | 0.569 | 29.6 | 84.6 |  | 51.700 | 18.2 | 87.1 |
|  | PDW | 0.589 | 65.4 | 48.8 |  | 0.604 | 85.2 | 34.2 |  | 58.700 | 57.7 | 56.0 |

Abbreviations: PE, preeclampsia; PC, platelet count; MPV, mean platelet volume; PCT, plateletcrit; PDW, platelet distribution width; AUC, areas under the ROC.

**Table S3** Detection rates for preeclampsia＜37, ≥37 weeks of gestation, and for all preeclampsia for 5% and 10% false-positive rates based on maternal characteristics and platelet parameters 12-15 gestational weeks (n=6381)

|  | Preterm PE | | | |  | Term PE | | | |  | Total PE | | | |
| --- | --- | --- | --- | --- | --- | --- | --- | --- | --- | --- | --- | --- | --- | --- |
|  | AUC | *p*- value | Detection rate (95%) | |  | AUC | *p*- value | Detection rate (95%) | |  | AUC | *p*- value | Detection rate (95%) | |
|  |  |  | FPR 5% | FPR 10% |  |  |  | FPR 5% | FPR 10% |  |  |  | FPR 5% | FPR 10% |
| Base model | 0.777 |  | 23.5 (5.9-47.1) | 29.4 (5.9-52.9) |  | 0.762 |  | 22.0 (12.0-34.0) | 36.0 (22.0-50.0) |  | 0.759 |  | 19.4 (10.5-29.9) | 37.3 (25.4-47.8) |
| plus |  |  |  |  |  |  |  |  |  |  |  |  |  |  |
| PC | 0.792 | 0.671 | 29.4 (5.9-52.9) | 35.3 (11.8-58.8) |  | 0.759 | 0.788 | 24.0 (12.0-36.0) | 34.0 (24.0-50.0) |  | 0.758 | 0.968 | 25.4 (14.9-35.8) | 34.3 (23.9-46.3) |
| MPV | 0.791 | 0.592 | 23.5 (5.9-47.1) | 47.1 (23.5-70.6) |  | 0.785 | 0.150 | 22.0 (12.0-36.0) | 38.0 (26.0-52.0) |  | 0.765 | 0.476 | 17.9 (9.0-28.4) | 34.3 (22.4-46.3) |
| PCT | 0.780 | 0.781 | 23.5 (5.9-41.2) | 35.3 (11.8-58.8) |  | 0.779 | 0.270 | 26.0 (14.0-38.0) | 34.0 (22.0-48.0) |  | 0.773 | 0.210 | 22.4 (13.4-32.8) | 34.3 (23.9-46.3) |
| PDW | 0.780 | 0.860 | 23.5 (5.9-47.1) | 35.3 (11.8-58.8) |  | 0.768 | 0.482 | 24.0 (12.0-38.0) | 38.0 (24.0-52.0) |  | 0.760 | 0.921 | 23.9 (13.4-35.8) | 37.3 (26.9-49.3) |
| PC + MPV + PCT + PDW | 0.800 | 0.568 | 29.4 (11.8-52.9) | 47.1 (23.5-70.6) |  | 0.797 | 0.094 | 28.0 (16.0-42.0) | 38.0 (24.0-50.0) |  | 0.776 | 0.274 | 29.9 (19.4-40.3) | 38.8 (26.9-50.8) |

Multivariate logistic regression models was used to develop the base models with the recognized historic risk factors recommended by NICE/ ACOG, including maternal age (continuous), pre-pregnancy weight (continuous), height (continuous), cigarette smoking during pregnancy (yes or no), parity (multiparous or nulliparous), inter-pregnancy interval (continuous), way of conception (spontaneous or in-vitro fertilization), history of chronic hypertension (yes or no), history of pre-existing diabetes mellitus (yes or no), family history of chronic hypertension (yes or no). PE, preeclampsia; PC, platelet count; MPV, mean platelet volume; PCT, plateletcrit; PDW, platelet distribution width; AUC, areas under the ROC.

**Table S4** The incremental value of platelet parameters at 12-15 gestational weeks for preeclampsia prediction (n=6381)

|  |  | | NRI | *p*-value | Event NRI | *p*-value | Non-Event NRI | *p*-value | IDI | *p*-value | Relative IDI (%) |
| --- | --- | --- | --- | --- | --- | --- | --- | --- | --- | --- | --- |
| Total PE (n=67) | | |  |  |  |  |  |  |  |  |  |
|  | | Base model | Ref. |  | Ref. |  | Ref. |  | Ref. |  | Ref. |
|  | | Base model + PC | 0.382 | 0.002 | -0.070 | 0.541 | 0.460 | <0.0001 | 0.0039 | 0.002 | 34.95 |
|  | | Base model + MPV | 0.202 | 0.101 | -0.010 | 0.903 | 0.220 | <0.0001 | 0.0009 | 0.259 | 8.10 |
|  | | Base model + PCT | 0.365 | 0.003 | 0.580 | <0.0001 | -0.220 | <0.0001 | 0.0015 | 0.075 | 13.75 |
|  | | Base model + PDW | 0.215 | 0.080 | 0.130 | 0.272 | 0.080 | <0.0001 | 0.0012 | 0.100 | 10.48 |
|  | | Base model + PC + MPV + PCT + PDW | 0.453 | <0.001 | 0.280 | 0.020 | 0.170 | <0.0001 | 0.0081 | 0.001 | 73.35 |
| Preterm PE (n=17) | | |  |  |  |  |  |  |  |  |  |
|  | | Base model | Ref. |  | Ref. |  | Ref. |  | Ref. |  | Ref. |
|  | | Base model + PC | 0.535 | 0.028 | 0.180 | 0.467 | 0.360 | <0.0001 | 0.0022 | 0.161 | 44.17 |
|  | | Base model + MPV | 0.274 | 0.259 | -0.410 | 0.090 | 0.690 | <0.0001 | 0.0004 | 0.594 | 7.85 |
|  | | Base model + PCT | 0.105 | 0.665 | -0.530 | 0.029 | 0.630 | <0.0001 | 0.0003 | 0.513 | 5.75 |
|  | | Base model + PDW | 0.356 | 0.143 | -0.290 | 0.225 | 0.650 | <0.0001 | 0.0025 | 0.301 | 51.75 |
|  | | Base model + PC + MPV + PCT + PDW | 0.456 | 0.060 | 0.290 | 0.225 | 0.160 | <0.0001 | 0.0064 | 0.074 | 13.07 |
| Term PE (n=57) | | |  |  |  |  |  |  |  |  |  |
|  | | Base model | Ref. |  | Ref. |  | Ref. |  | Ref. |  | Ref. |
|  | | Base model + PC | 0.335 | 0.018 | -0.120 | 0.396 | 0.450 | <0.0001 | 0.0022 | 0.011 | 28.10 |
|  | | Base model + MPV | 0.305 | 0.032 | -0.360 | 0.011 | 0.660 | <0.001 | 0.0015 | 0.159 | 19.45 |
|  | | Base model + PCT | 0.454 | 0.001 | 0.520 | <0.001 | -0.070 | <0.0001 | 0.0019 | 0.043 | 24.87 |
|  | | Base model + PDW | 0.2302 | 0.105 | 0.080 | 0.5716 | 0.150 | <0.0001 | 0.0004 | 0.473 | 5.34 |
|  | | Base model + PC + MPV + PCT + PDW | 0.586 | <0.0001 | 0.440 | 0.002 | 0.150 | <0.0001 | 0.0058 | 0.002 | 75.25 |

Multivariate logistic regression models was used to develop the base models with the recognized historic risk factors recommended by NICE/ ACOG, including maternal age (continuous), pre-pregnancy weight (continuous), height (continuous), cigarette smoking during pregnancy (yes or no), parity (multiparous or nulliparous), inter-pregnancy interval (continuous), way of conception (spontaneous or in-vitro fertilization), history of chronic hypertension (yes or no), history of pre-existing diabetes mellitus (yes or no), family history of chronic hypertension (yes or no). PE, preeclampsia; PC, platelet count; MPV, mean platelet volume; PCT, plateletcrit; PDW, platelet distribution width; NRI, net reclassification improvement; IDI, integrated discrimination improvement.

**Table S5** Platelet parameters of pregnant women with multiple measurements during 16-19 gestational weeks.

| **Platelet parameters** | **First measurement (n=243)** | **Last measurement**  **(n=243)** | ***p*-value** |
| --- | --- | --- | --- |
| Test weeks, wk (mean ± SD) | 17.0 ± 0.79 | 18.6 ± 0.96 | **<0.001** |
| Platelet count, ×10^9^/L (mean ± SD) | 245.9 ± 60.1 | 245.1 ± 61.2 | 0.735 |
| Mean platelet volume, fL (mean ± SD) | 9.64 ± 1.41 | 9.66 ± 1.40 | 0.730 |
| Plateletcrit, % (mean ± SD) | 0.236 ± 0.061 | 0.235 ± 0.064 | 0.711 |
| Platelet distribution width, fL (mean ± SD) | 13.26 ± 2.64 | 13.41 ± 2.71 | 0.280 |


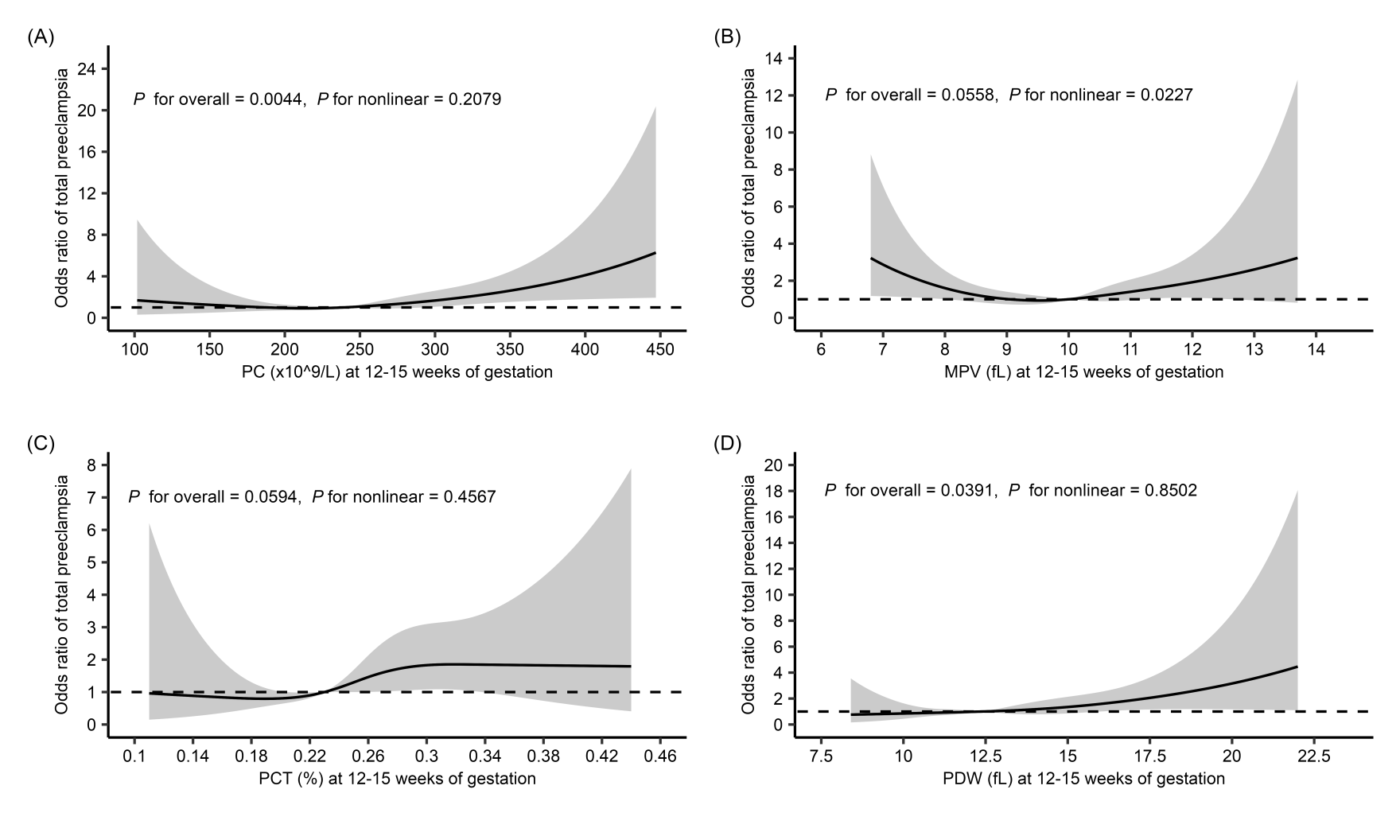


**Figure S1** Associations between platelet parameters during 12-15 weeks of gestation and preeclampsia with a restricted cubic spline function. Risk of preeclampsia/eclampsia in relation to (A) platelet count (PC), (B) mean platelet volume (MPV), (C) plateletcrit (PCT), and (D) platelet distribution width (PDW). Platelet parameters were fitted using a restricted cubic spline function. The median values of platelet parameters were used as the referent, with PC at 250 × 109/L, MPV at 10.0 fL, PCT at 0.23%, and PDW at 13.0 fL. The overall p value tested the relationships between platelet parameters and the risk of preeclampsia, including linear and non-linear relationships, and the non-linear p value tested the non-linear relationships. Odds ratios are represented by the solid line and the 95% confidence interval by the shaded. PE, preeclampsia; PC, platelet count; MPV, mean platelet volume; PCT, plateletcrit; PDW, platelet distribution width.

.

**
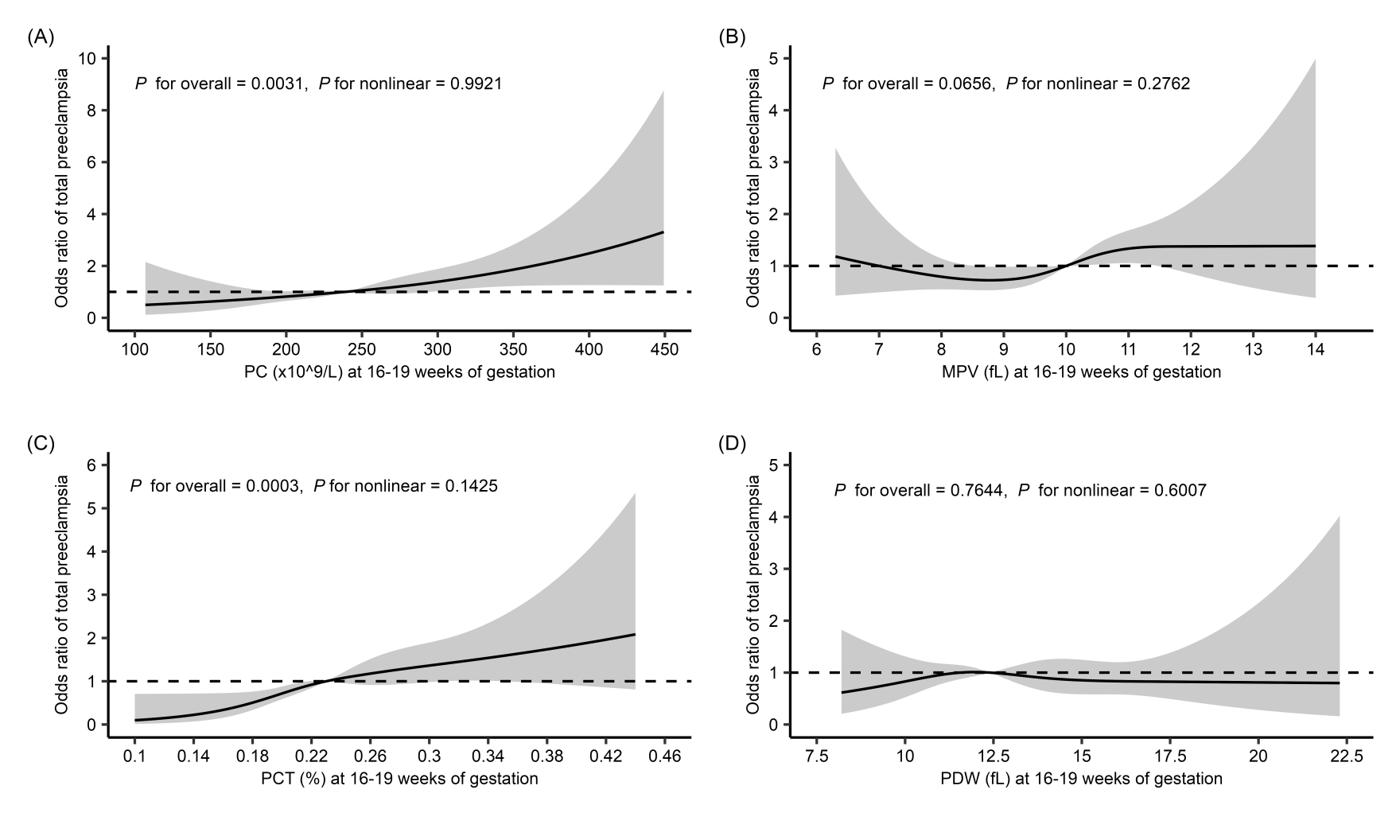
**

**Figure S2** Associations between platelet parameters during 16-19 weeks of gestation and preeclampsia with a restricted cubic spline function. Risk of preeclampsia/eclampsia in relation to (A) platelet count (PC), (B) mean platelet volume (MPV), (C) plateletcrit (PCT), and (D) platelet distribution width (PDW). Platelet parameters were fitted using a restricted cubic spline function. The median values of platelet parameters were used as the referent, with PC at 239 × 109/L, MPV at 10.0 fL, PCT at 0.23%, and PDW at 12.4 fL. The overall p value tested the relationships between platelet parameters and the risk of preeclampsia, including linear and non-linear relationships, and the non-linear p value tested the non-linear relationships. Odds ratios are represented by the solid line and the 95% confidence interval by the shaded. PE, preeclampsia; PC, platelet count; MPV, mean platelet volume; PCT, plateletcrit; PDW, platelet distribution width.
